# No Easy Way Out for EZH2: Its Pleiotropic, Noncanonical Effects on Gene Regulation and Cellular Function

**DOI:** 10.3390/ijms21249501

**Published:** 2020-12-14

**Authors:** Jun Wang, Gang Greg Wang

**Affiliations:** 1 Lineberger Comprehensive Cancer Center, University of North Carolina at Chapel Hill School of Medicine, Chapel Hill, NC 27599, USA; jun113@email.unc.edu; 2 Department of Biochemistry and Biophysics, University of North Carolina at Chapel Hill School of Medicine, Chapel Hill, NC 27599, USA

**Keywords:** chromatin, histone, EZH2, methylation, Polycomb repressive complex, coactivator, gene transcription, cancer

## Abstract

Enhancer of zeste homolog 2 (EZH2) plays critical roles in a range of biological processes including organ development and homeostasis, epigenomic and transcriptomic regulation, gene repression and imprinting, and DNA damage repair. A widely known function of EZH2 is to serve as an enzymatic subunit of Polycomb repressive complex 2 (PRC2) and catalyze trimethylation of histone H3 lysine 27 (H3K27me3) for repressing target gene expression. However, an increasing body of evidence demonstrates that EZH2 has many “non-conventional” functions that go beyond H3K27 methylation as a Polycomb factor. First, EZH2 can methylate a number of nonhistone proteins, thereby regulating cellular processes in an H3K27me3-independent fashion. Furthermore, EZH2 relies on both methyltransferase-dependent and methyltransferase-independent mechanisms for modulating gene-expression programs and/or epigenomic patterns of cells. Importantly, independent of PRC2, EZH2 also forms physical interactions with a number of DNA-binding factors and transcriptional coactivators to context-dependently influence gene expression. The purpose of this review is to detail the complex, noncanonical roles of EZH2, which are generally less appreciated in gene and (epi)genome regulation. Because EZH2 deregulation is prevalent in human diseases such as cancer, there is increased dependency on its noncanonical function, which shall have important implications in developing more effective therapeutics.

## 1. The Canonical Role of EZH2 as a Polycomb Factor Mediating H3K27 Methylation

**Figure 1:**
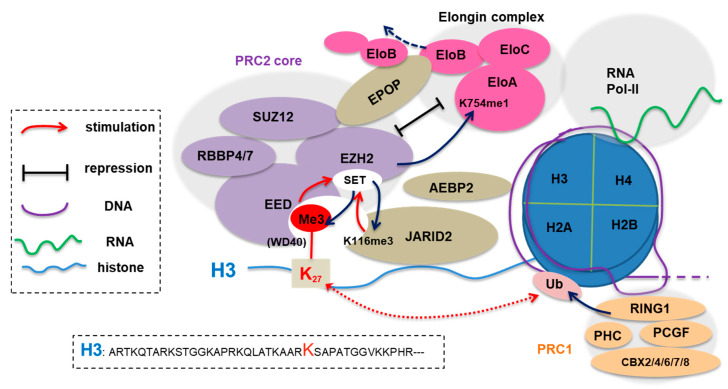
Enhancer of zeste homolog 2’s (EZH2) canonical function as enzymatic subunit of Polycomb repressive complex 2 (PRC2), which additionally consists of embryonic ectoderm development (EED), suppressor of zeste 12 homolog (SUZ12), and retinoblastoma-binding protein 7/4 (RBBP7/4). EZH2:PRC2 silences transcription at least partly through trimethylation of histone H3 lysine 27 (H3K27me3). PRC1, composed of the PCGF (such as BMI1), RING1, CBX (one of CBX2/4/6/7/8) and PHC subunits, both induces histone H2A lysine-119 mono-ubiquitination (H2AK119ub1) and mediates chromatin compaction via a phase separation-related mechanism [25,26]. Besides inducing H3K27me3, EZH2:PRC2 also methylates its own subunit/cofactors such as EZH2 and JARID2 for PRC2 activation. In particular, both H3K27me3 and JARID2-K116me3 are “recognized” by the WD40 repeat module of EED, inducing allosteric activation and possibly on-chromatin spreading of PRC2. Additionally, via the EPOP-mediated sequestration of EloB/C and PRC2:EZH2-mediated methylation of elongin-A (EloA), PRC2 also suppresses assembly and/or activity of the Elongin complex, thus contributing to suppression of target gene expression.

Polycomb group (PcG) genes were initially identified as regulator of body segmentation and repressor of *Hox* gene expression in *Drosophila* and were subsequently shown to be conserved among other organisms such as mammal. Biochemical charactarizations of Polycomb repressive complexes 2 and 1 (PRC2 and PRC1) revealed their specific histone-modifying activities (Figure 1) [1,2,3,4,5,6,7,8]. An active PRC2 “core” complex is composed of at least four subunits— enhancer of zeste homolog 2 (EZH2) or related EZH1, embryonic ectoderm development (EED), suppressor of zeste 12 homolog (SUZ12), and retinoblastoma-binding protein 7 (RBBP7) or related RBBP4. It is well established that EZH2/1 functions as PRC2’s catalytic subunit to repress gene transcription at least partly through inducing trimethylation of histone H3 lysine 27 (H3K27me3), a chromatin mark that can serve as a docking site for recruiting “effector/reader” proteins. For example, H3K27me3 is “recognized” by the WD40-repeat domain of EED (Figure 1), thereby forming a feedforward loop for deposition and spreading of H3K27me3 in the genome [9]; importantly, H3K27me3 is also engaged by distinctive classes of “reader” domains—the conserved chromodomain (CD) of the CBX subunit (CBX2/4/6/7/8; Figure 1) in PRC1 [10,11,12], as well as the BAH module harbored within several chromatin-associated proteins in eukaryotes including plant [13,14,15], fungus [16] and animal [17], all of which contribute to Polycomb gene silencing potentially through different molecular mechanisms that are still under investigation. Thus, EZH2, an integral component of PRC2, mediates gene silencing, a process implicated in many biological processes such as cell fate decision-making, cell lineage specification, and oncogenesis. Readers shall refer to other reviews [18,19,20,21,22,23,24] on the topic of Polycomb proteins.

## 2. Introduction to EZH2′s Noncanonical Roles Beyond Polycomb and H3K27me3

Despite EZH2′s well-established function as part of PRC2 for mediating H3K27me3 and gene repression, accumulating evidence demonstrates non-canonical roles of EZH2. These noncanonical activities are mainly due to (i) EZH2-mediated nonhistone substrate methylation, (ii) EZH2-mediated transcriptional activation instead of repression, (iii) a scaffolding function of EZH2 in binding to protein or RNA, and (iv) PRC2-independent interactions of EZH2 with a set of transcriptional (co)activators. Indeed, cases exist where EZH2′s catalytic inhibition does not phenocopy or even remotely resemble knockdown or knockout of EZH2 under a range of biological contexts [27,28,29,30]. For example, through a methyltransferase-independent mechanism, EZH2 induces transcriptional activation of RelB, a noncanonical NF-kB factor, to sustain tumor phenotypes in triple-negative breast cancer (TNBC) [30]. Certain intrinsically-disordered regions within EZH2 were recently shown to harbor transcriptional activation activity [31,32].

Overall, the existing literature suggests EZH2’s noncanonical roles to be more evident in cancer where EZH2 frequently shows overexpression due to various genomic alterations, such as genomic loss of EZH2-targeting miRNAs [33,34]. It is conceivable that an increased level of EZH2 may promote “nonconventional” protein–protein interactions unrelated to PRC2. Additionally, cellular contexts such as kinase signaling may profoundly alter PRC2 functionality. In the next sections, we will discuss about nonhistone methylation and noncanonical effects by EZH2.

## 3. Nonhistone Substrate Methylation by EZH2

Lysine methylation is a posttranslational modification (PTM) known to modulate protein function and protein–protein interaction. While EZH2 (or related EZH1) is known as sole methyltransferase in mediating low-to-high degrees of H3K27 methylation, increasing evidence supports that EZH2 also induces lysine methylation beyond histones and on various nonhistone substrates, many of which are transcription factors (TFs) and chromatin-associated proteins (Table 1). Please note that nonhistone substrate methylation by EZH2 can be executed in the PRC2-dependent or PRC2-independent fashion, and that the biological consequences of these nonhistone methylations are also context-dependent.

### 3.1. Methylation of EZH2, JARID2 and Elongin-A (EloA) by PRC2:EZH2 Is Associated with Gene Silencing

#### 3.1.1. JARID2

JARID2, an accessory cofactor of PRC2, regulates the chromatin targeting and enzymatic activity of PRC2. JARID2 carries a H3-mimicking sequence and, in addition to H3, PRC2 also trimethylates JARID2 at lysine 116 (JARID2-K116me3; Figure 1 and Table 1). Akin to H3K27me3, JARID2-K116me3 is bound by the WD40 domain of EED, an event crucial for the allosteric activation and chromatin spreading of PRC2 [35,47].

#### 3.1.2. EZH2

**Figure 2:**
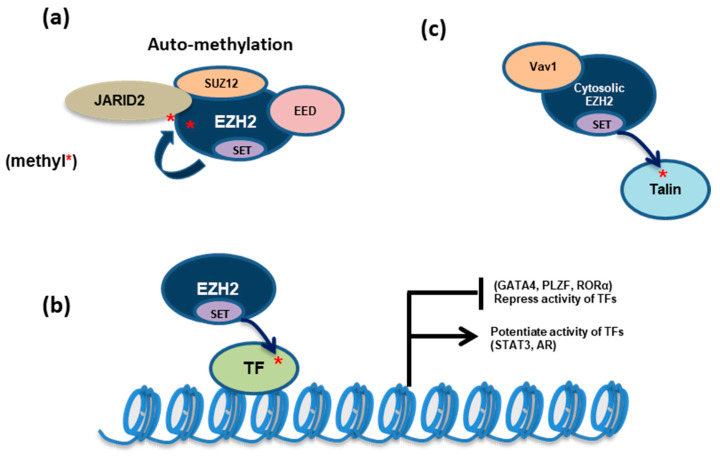
Besides inducing H3K27me3, EZH2 also methylates nonhistone proteins, either in the PRC2-dependent or PRC2-independent manner. (**a**) PRC2:EZH2 methylates EZH2 (automethylation) or JARID2, a PRC2 accessory cofactor, for enhancing PRC2:EZH2’s methyltransferase activity. (**b**) EZH2 can methylate a number of transcription factors (TFs) to either activate or repress their transcription-regulatory function. (**c**) In the cytosol, EZH2 interacts with Vav1 and methylates talin, thereby regulating cell adhesion and mobility. *, EZH2-methylated lysine site.

PRC2 also automethylates EZH2 at the lysines 510, 514, and 515 [36,37]. These three residues reside within a disordered but highly conserved loop between so-called SANT2L and CXC domains of EZH2 and can be in close proximity to the substrate-binding pocket of the catalytic (SET) domain. K510 and K514 are the major sites of EZH2 automethylation whereas K515 automethylation occurs concomitantly with that of K514. Furthermore, EZH2 automethylation of K510 and K515 was detected only in the presence of K514 methylation *in cis*, suggesting that the latter represents a prerequisite for the other automethylation events. Functional studies indicated EZH2 automethylation acting as a self-activating mechanism in the absence of stimulatory cofactors such as AEBP2 (Figure 2a and Table 1). Automethylated EZH2 in PRC2 not only exhibits the increased histone methyltransferase activity but is also required for attaining proper H3K27me3 levels in cells [36,37]. Occurring independently of its chromatin recruitment, PRC2 automethylation was reported to promote its accessibility to the histone substrate [36,37].

#### 3.1.3. Elongin A (EloA)

EloA (Figure 1), together with EloB/C, forms the Elongin complex to promote transcriptional elongation by RNA polymerase II (Pol-II), an integral part of gene activation [48]. A recent study shows that PRC2 monomethylates the lysine 754 of EloA (EloA-K754me1; Table 1) and this methylation is important for tuning down the transcription of PRC2 target genes [38]. In the mouse embryonic stem cells (ESCs), the mutation of the EloA site that prevents lysine methylation by PRC2 leads to upregulation of a subset of PRC2 targets, which then interferes with differentiation potentials of ESCs [38]. More interestingly, EPOP, a recently identified cofactor of PRC2 [49,50], also interacts with EloB/C, providing a second connection between the PRC2 and Elongin complexes. Here, biochemical interactions of EloB/C with EloA and with EPOP:PRC2 are mutually exclusive (Figure 1) [50] and, in mouse ESCs, co-targeting of these two functionally antagonizing complexes to same genes establishes a poised chromatin state reminiscent of so-called “bivalent domain” genes [51,52]. Altogether, the PRC2:EPOP complex both methylates EloA and competes with it for EloB/C, thereby downregulating gene expression.

### 3.2. Methylation of Transcription Factors (TFs) by EZH2 May Either Enhance or Repress Their Respective Gene-Regulatory Activities

#### 3.2.1. Globin Transcription Factor 4 (GATA4)

GATA4 is a key dosage-sensitive regulator of heart development [43]. Part of the effect by this cardiac TF is via recruitment of histone acetyltransferase p300 to specific chromatin loci for promoting gene transcription. EZH2 directly interacts with and methylates GATA4 at its lysine 299, an event that reduces the GATA4:p300 interaction and thus attenuates GATA4 function. This work establishes a new mechanism underlying the PRC2-related gene repression, in which PRC2 directly methylates a transcriptional activator to suppress its activity (Figure 2b and Table 1) [43].

#### 3.2.2. STAT3

Glioblastoma multiforme (GBM) displays cellular hierarchies, harboring a subpopulation of glioblastoma stem cells (GSCs). In GSCs rather than non-stem bulk tumor, AKT induces EZH2 phosphorylation at Ser21 and this so-called “phospho-switch” of EZH2 (see Section 4) enhances its interaction with STAT3, which is followed by STAT3 lysine-180 trimethylation by EZH2, resulting in increased tyrosine phosphorylation and activation of STAT3 [39]. Thus, this AKT-EZH2-STAT3 pathway is critical for the maintenance of GSCs and GBM tumorigenicity [39]. Similarly, in response to IL6, dimethylation of STAT3 at lysine 49 by EZH2 also promotes transcription of STAT3 targets in colon cancer [40]. In both cases, STAT3 methylation by EZH2 is important for STAT3 activation, which context-dependently potentiates the oncogenic gene-expression program (Figure 2b and Table 1).

#### 3.2.3. Retinoic Acid-Related Orphan Nuclear Receptor α (RORα)

RORα was suggested to function as a tumor suppressor—activation of RORα attenuates migratory and invasive features of androgen-independent prostate cancer, reduces WNT/β-catenin signaling in colon cancer, and suppresses proliferation of breast cancer cells [53]. EZH2 was shown to mono-methylate RORα at lysine 38 [45]. Such monomethylated RORα is then recognized by the DCAF1/DDB1/CUL4 E3 ubiquitin ligase complex and undergoes ubiquitination and degradation. Thus, EZH2 creates a “methyl degron” at a nonhistone substrate (RORα), linking it to the methylation-dependent ubiquitination machinery for degradation (Figure 2b and Table 1).

#### 3.2.4. Promyelocytic Leukemia Zinc Finger Protein (PLZF)

PLZF acts to finetune the balance between self-renewal and differentiation of hematopoietic stem/progenitor cells, partly through binding to chromatin-modifying factors. PLZF was known to have dual effect in both transcriptional repression and activation. Acting in a PRC2-independent fashion, EZH2 directly methylates PLZF leading to its ubiquitinylation and degradation (Figure 2b and Table 1), which then impacts on development of natural killer T cells and homeostasis of the immune system [44]. Moreover, another study showed that, in the KG-1 CD34+ myeloblastic hematopoietic cells, EZH2 interacts with PLZF and co-regulates a set of active genes that are marked by the active histone mark H3K4me3 and lack binding of PRC2 (SUZ12) or H3K27me3 [54]. These findings highlighted a complicated interplay between EZH2 and PLZF under different contexts.

#### 3.2.5. β-Catenin

Wnt/β-catenin signaling is required for ESC pluripotency by inducing mesodermal differentiation and inhibiting neuronal differentiation. Hoffmeyer et al. showed that β-catenin is trimethylated (β-catMe3) at lysine 49 by EZH2, and such a β-catMe3 form acts as transcriptional corepressor of *sox1* and *sox3*, the differentiation regulators known to be crucial for differentiation of ESCs into neuronal or mesodermal progenitor cell lineages [55].

### 3.3. Methylation of Proliferating Cell Nuclear Antigen (PCNA) by EZH2 Promotes DNA Replication

During cell replication, PCNA, in the form of a ring-shaped homotrimer complex, promotes DNA replication via binding to DNA polymerase. EZH2 interacts with PCNA via the PIP box and dimethylates PCNA at lysine 110 (Table 1). Such dimethylation of PCNA is essential for stabilization of the PCNA trimer and binding of DNA polymerase δ to PCNA, indicating a role of EZH2 in orchestrating the genome duplication [41].

## 4. The “Phospho-Switch” Model Suggests an Involvement of Kinase Signaling for Functional Switch of EZH2, from a Canonical Gene-Repressive Role to a Noncanonical Gene-Activation One

Increasing evidence suggests EZH2 dynamically regulated by cellular and environmental cues, which underlies EZH2’s functional switch from PRC2 to a noncanonical activity. For the latter, EZH2 harbors the hidden, partially disordered transactivation domain (TAD) capable of directly interacting with the coactivator and acetyltransferase p300 to activate gene expression in a p300-dependent manner [31,32].

### 4.1. AKT-Mediated Phosphorylation of EZH2 at Serine 21 (EZH2-S21phospho)

**Figure 3:**
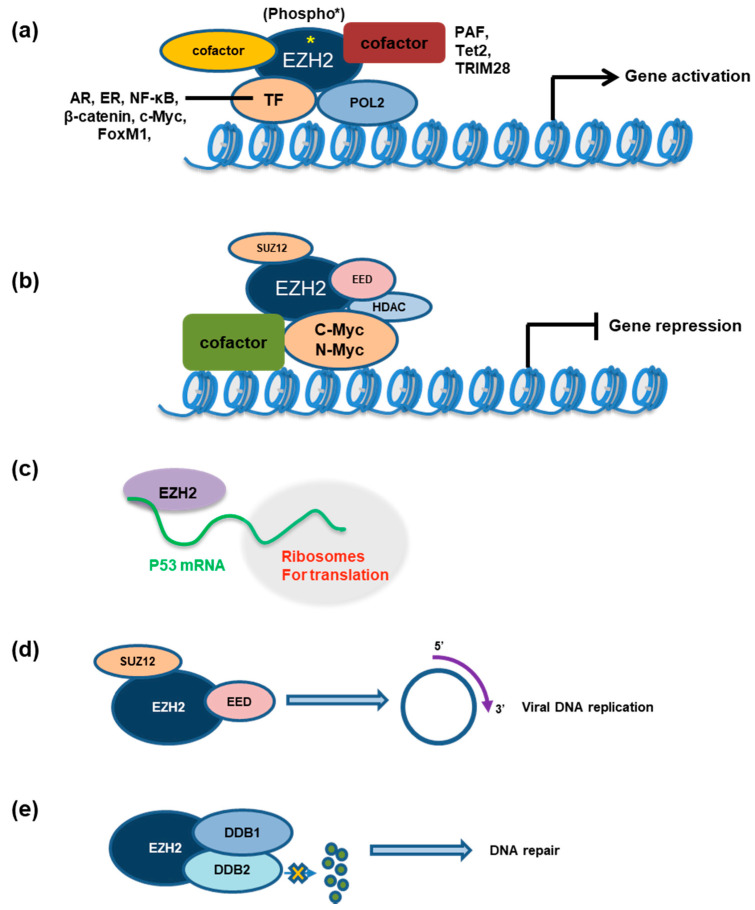
EZH2 establishes a range of canonical (in form of PRC2) and noncanonical protein–protein interactions (such as AR and NF-kB), regulating gene expression and other biological processes. (**a**) EZH2, likely acting as a scaffold protein, functions as coactivator with transcription factors and RNA Pol-II for inducing target gene activation. It was shown that certain cellular contexts, such as JAK3-mediated EZH2 phosphorylation at Tyr-244, TGFβ1-induced tissue damage, and viral infection, all facilitate the release of EZH2 from PRC2 to establish a noncanonical complex with TFs (such as AR and NF-kB), RNA Pol-II, and coactivators (such as PAF) for transcriptional activation of target genes. *, phosphorylation. (**b**) C-Myc or N-Myc interacts with EZH2 and recruits PRC2 to its targets for gene repression. (**c**) EZH2 binds to mutant P53 mRNA in cancer, promoting its stability and cap-independent protein translation. (**d**) EZH2 promotes replication of human cytomegalovirus (HCMV) after cellular infection. (**e**) EZH2 forms complex with DDB1 and DDB2, stabilizing the latter to promote DNA damage repair.

AKT kinase activation is prevalent in human cancer, owing to the loss-of-function (LOF) mutation of PTEN or activation mutation of upstream PI3K. H3K27me3 by PRC2:EZH2 is globally suppressed by activated AKT, which directly phosphorylates EZH2 at serine 21 (EZH2-S21phospho) [56]. It was shown that EZH2-S21phospho exerts a “neomorphic” effect in cancer by switching EZH2’s role from PRC2-associated repressor to coactivator [39,42]. For example, EZH2-S21phospho may reduce EZH2 binding to histone H3 [56] and enhance its interactions to non-PRC2 partners, such as androgen receptor (AR) in advanced prostate tumor and STAT3 in GBM (Figure 3a and Table 2).

### 4.2. JAK-Mediated Phosphorylation of EZH2 at Tyrosine 244 (EZH2-Y244phospho)

Moreover, it was shown that, in natural killer/T-cell lymphoma (NKTL) models, phosphorylation of EZH2 at Y244 by JAK3 causes EZH2 dissociation from PRC2 and enhances its association with RNA Pol-II. Such a switch from transcriptional corepressor to coactivator led to upregulation of a set of genes involved in DNA replication, cell cycle progression, biosynthesis, stemness and invasiveness [57] (Figure 3a). In support of the involvement of EZH2 phosphorylation, pharmacological inhibition of JAK3, but not inhibitors targeting EZH2’s methyltransferase activity, can reduce malignant growth of NKTL cells [57]. Notably, JAK activation also exerts gene-regulatory effects via direct phosphorylation of histone H3 Y41 (H3Y41) in cancer [58,59,60]. Primary and secondary effects are likely involved in interplays among these factors, which remain to be further defined.

## 5. EZH2 Forms Interactions with TFs Crucial for Gene-Expression Regulation 

### 5.1. EZH2 Interacts with Androgen Receptor (AR), Enhancing AR Signaling in Advanced Prostate Cancer

ChIP-seq-based mapping of EZH2 binding and H3K27me3 in LNCaP-abl cells, a castration-resistant prostate cancer model, showed that approximately 90% of EZH2-bound regions overlap the expected H3K27me3; however, the remaining ~10% of so-called EZH2-“solo” peaks were found enriched in active histone marks (H3K4me2 and H3K4me3) and RNA Pol II, suggesting a potential role in gene activation [42]. As mentioned above, PTEN loss and resultant AKT activation is common during prostate cancer progression. In agreement with an AKT-mediated phosphorylation [56] and molecular “switch” of EZH2, Ser21-phosphorylated EZH2 interacts with AR [42]. EZH2 likely mediates AR methylation as well because knocking down EZH2 decreased AR methylation, which could not be rescued with the catalytic-domain-deleted EZH2. Additionally, EZH2 and AR are both required for transcription of their co-targeted genes (Figure 3a and Table 2), which depends on intact methyltransferase activity of EZH2 [42]. A separate study further showed that EZH2 also activates transcription of the AR gene itself through direct occupancy at its promoter and that this activating role of EZH2 is independent of PRC2 and its methyltransferase activity [61]. These works delineated a multilevel crosstalk between EZH2 and activated AR signaling during prostate tumorigenesis.

### 5.2. EZH2 Interacts with Myc to Promote Oncogenic Gene-Expression Programs in Both PRC2-Dependent and PRC2-Independent Manners

Several studies have shown that Myc family proteins such as C-Myc and N-Myc (Figure 3b and Table 2) directly interact with EZH2, which leads to recruitment of EZH2 and/or PRC2 to Myc target loci for gene silencing [62,63,64,65,66,67]. However, under different contexts, EZH2 and C-Myc can also cooperate in activating gene transcription. For example, EZH2 directly binds to the IGF1R promoter along with C-Myc and upregulates IGF1R expression in a subgroup of chronic lymphocytic leukemia, resulting in activation of the downstream PI3K [68].

### 5.3. EZH2 Context-Dependently Interacts with Estrogen Receptor (ER) and Nuclear Factor-Kappa B (NF-κB) among Different Breast Cancer Subtypes

Interestingly, in breast cancer, EZH2 can act as a coactivator via two different mechanisms, depending on the presence or absence of ER. First, in ER-positive luminal-like breast cancer cells, EZH2 was shown to have a transcriptional activation role through physical interactions with ER and β-catenin (Figure 3a and Table 2) [32]. Here, EZH2 was found to transactivate a set of transcripts commonly regulated by estrogen and WNT signaling such as c-Myc and cyclin D1, thereby promoting cell cycle progression. Moreover, in a transgenic mouse model, EZH2 interaction with β-catenin was shown to be critical for promoting nuclear accumulation and activation of the latter in mammary epithelial cells [69]. It shall be noted that EZH2 also associates with ER and represses expression of NF-κB targets by inducing H3K27me3 on their promoters [70], which operates in a canonical fashion.

In contrast, in the ER-negative basal-like breast cancer cells, EZH2 interacts with RelA and RelB (Figure 3a and Table 2), two core subunits of NF-κB, and functions as NF-κB coactivator independent of its histone methyltransferase activity [70]; this role of EZH2 in promoting NF-κB signaling was in agreement with the EZH2-mediated transcriptional activation of RelB, again through a methyltransferase-independent mechanism, to sustain the self-renewal and tumor-initiating phenotypes of TNBC cells [30]. Thus, among different breast cancer types, EZH2 acts as a double-faceted molecule, either as coactivator or corepressor of NF-κB targets, depending on the cellular context.

### 5.4. A β-Catenin:PAF:EZH2 Complex Activates WNT Signaling in Colon Cancer

In colon cancer cells, the PCNA-associated factor (PAF) and EZH2 were shown to induce hyperactivation of WNT signaling [71]. Upon WNT signaling activation, PAF dissociates from PCNA and directly binds β-catenin; PAF (Figure 3a and Table 2) also recruits EZH2 to form a β-catenin:PAF:EZH2 transcriptional complex, activating WNT target genes. Interestingly, the gene-activation effect by EZH2:PAF on β-catenin targets does not require EZH2’s methyltransferase activity, indicative of a scaffold role [71]. Please note that this interaction of EZH2 and β-catenin differs from what was observed in ESCs as discussed in the above ‘’Section 3.2.5’’, highlighting a context-dependent regulation that merits additional study.

### 5.5. In Response to Hypoxia, EZH2 Associates with Hypoxia-Inducible Factor 1, α-Subunit (HIFI-α) to Promote Expression of Invasion-Related Genes in Breast Tumor

HIFI-α is a crucial modulator of PRC2/EZH2 in breast cancer. In TNBC cells, high HIFI-α positively correlates with high EZH2 expression but low PRC2 activity [72]. In the absence of HIFI-α activation, EZH2:PRC2 mainly functions as a repressor to silence expression of matrix metalloproteinase genes (MMPs). HIF1-α induction upon hypoxia results in PRC2 inactivation by selective suppression of the expression of SUZ12 and EED, and then, EZH2 forms interaction with Forkhead box M1 (FoxM1) to promote the expression of MMPs, thus establishing a functional switch from PRC2(EZH2) towards the EZH2:FoxM1-dependent activation of genes related to tumor cell invasion (Figure 3a and Table 2) [72].

### 5.6. EZH2 Binds the Mutated p53 mRNA, Promoting Its Stability and Cap-Independent Translation

Zhao et al. recently showed that EZH2 binds to an internal ribosome entry site (IRES) in the 5’UTR of both wild-type and mutated p53 mRNAs, and increases mRNA stability and cap-independent protein translation in a methyltransferase-independent manner (Figure 3c) [73]. EZH2 thus augments the p53 gain-of-function mutant-mediated cancer growth and metastasis by increasing the level of mutant p53 proteins.

### 5.7. EZH2 and TRIM28 Interact for Regulating Tumor Progression

Tripartite motif-containing protein 28 (TRIM28; also known as KRAB-associated protein 1 or KAP1) was previously shown to promote breast cancer proliferation and metastatic progression and required for maintenance of mouse ESC pluripotency and self-renewal [74,75]. In the MCF7 ER-positive breast cancer cells, EZH2 interacts with subunits of the SWI/SNF chromatin remodeler complex and TRIM28 (Figure 3a and Table 2), forming a complex distinct from PRC2. In this context, EZH2 and TRIM28 co-activate a set of “stemness” genes that promote mammosphere formation [76]. A recent work also showed that the Kruppel-associated box (KRAB) domain, a module within the KRAB zinc finger (KRAB-ZF) proteins required for TRIM28 recruitment and subsequent repression of transposable elements by TRIM28-associated corepressor complexes, was able to inhibit TRIM28:EZH2 interaction and caused degradation of TRIM28, EZH2, and other PRC2 components, leading to a decreased H3K27me3 level in MCF7 cells [77]. TRIM28:EZH2 interactions appear complex and need additional investigation.

## 6. EZH2 Functions Go Beyond Polycomb and Gene Silencing

As above mentioned, EZH2 is critically involved in various DNA-templated processes such as gene transcription and DNA replication. In this section, we cover some additional biological processes regulated by EZH2.

### 6.1. EZH2 Contributes to Viral Infection Processes

Chromatin-based modification and silencing of viral genomes are crucial for dictating the outcome of viral infection. Recent studies reported that human cytomegalovirus (HCMV) replication depends on a noncanonical function of Polycomb complexes [28,78]. HCMV infection strongly induced upregulation of various PRC1 and PRC2 core components, such as EZH2, EED, SUZ12, and RING1B. Consequently, re-localization of PRC1/2 components but not H3K27me3 to viral replication compartments directly contributes to viral DNA replication, because knockdown of these polycomb proteins resulted in a significant reduction of newly synthesized viral particles. Small molecules solely inhibiting the enzymatic activity of PRC1/2 had no antiviral effects, supporting a noncanonical function of these Polycomb factors [28,78] (Figure 3d).

Likewise, during chronic hepatitis B virus (HBV) infection, a known major risk factor for developing hepatocellular carcinoma, EZH2’s noncanonical function is also involved [79]. Here, in animal liver tumors modeling HBV-mediated hepatocarcinogenesis, disease progression was found accompanied with SUZ12 downregulation and concurrent upregulation of PRC2 target genes, such as EpCAM and DLK1, which are normally silenced in uninfected hepatocytes. Fan et al. reported that loss of SUZ12 resulted in DNA demethylation of CpG dinucleotides within the regulatory elements (CpG island) associated with EpCAM and DLK1; and such DNA demethylation involves a noncanonical complex composed of RelA (a NF-κB complex subunit), EZH2, TET2 (a DNA demethylase), and inactive DNMT3L. This study supports a noncanonical role of EZH2 in mediating active DNA demethylation during oncogenic transformation [79].

### 6.2. EZH2 Is Involved in Cellular Response to DNA Repair, Contributing to Drug Resistance

In small cell lung cancer (SCLC), Koyen et al. reported that EZH2 has a non-catalytic, PRC2-independent role in stabilizing DDB2, a DNA repair factor, to promote nucleotide excision repair (NER) and cisplatin resistance in SCLC (Figure 3e) [80]. In SCLC cells subjected to the cisplatin-based chemotherapy, EZH2 complexes with the DDB1-DDB2 heterodimer and promotes DDB2 stability by impairing its ubiquitination, thereby facilitating DDB2 localization to cyclobutene pyrimidine dimer crosslinks for their repair [80].

### 6.3. EZH2 Functions to Maintain Organ Homeostasis During Tissue Damage

To understand an organ’s regenerative capacity and the aberrant repair process in diseased settings, a recent study showed that transforming growth factor β1 (TGFβ1)-damaged epithelium initiates a bidirectional fibrotic cascade within the mesenchyme [81]. On one hand, TGFβ1-induced damage facilitates the release of EZH2 from PRC2 to establish a novel fibrotic transcriptional complex composed of EZH2, RNA Pol-II, and nuclear actin for transcription activation of pro-fibrotic genes (Figure 3a); on the other hand, the liberation of EZH2 from PRC2 is accompanied by an EZH2-to-EZH1 switch within PRC2 to maintain H3K27me3 at TGFβ1 non-target genes [81]. The molecular underpinning of such a change involves a “phospho-switch” of EZH2, which merits more investigation.

### 6.4. Shuffling Between Nuclear and Cytosolic EZH2

It has been shown that EZH2 exists in the cytosol where it forms a complex with SUZ12 and EED and that cytosolic EZH2 methyltransferase activity is necessary for receptor-mediated signaling and actin reorganization in T lymphocytes and fibroblasts [82,83]. Here, cytosolic EZH2 methyltransferase complex in T cells was found to associate with the Rho GDP-GTP exchange factor, Vav1 [82,83], and to directly trimethylate Talin (Figure 2c and Table 1), a key regulator of cell migration, at its lysine 2454 [46]. EZH2-mediated methylation of Talin disrupts its binding to F-actin, resulting in a misregulated cell adhesion dynamic. As a support, disrupting the interaction of EZH2 with Vav1 abolished the regulatory effect of EZH2 on cell adhesion [46]. Consistently, EZH2 also plays a role in regulating integrin signaling and adhesion dynamics of neutrophils and dendritic cells [46]. EZH2 deficiency impairs the integrin-dependent transendothelial migration of innate leukocytes.

In ER-negative breast cancer, the p38 MAP kinase induces EZH2 phosphorylation at T367, an event critical for promoting EZH2 localization to the cytosol where it binds vinculin and other cytoskeletal proteins during the regulation of cell migration and invasion [84]. Overexpression of a phospho-T367-deficient mutant of EZH2 (T367A) inhibits its cytoplasmic localization and disrupts EZH2 interaction with cytoskeletal proteins, hence reducing the EZH2-related cancer cell invasion and spontaneous metastasis [84]. Likewise, cytoplasmic EZH2 was also reported to be lowly expressed in benign prostate epithelial cells but highly expressed in prostate cancer. Here, knockdown of EZH2 in the PC3 prostate tumor model enhanced polymerization of F-actin and formation of actin-rich filaments [85]. EZH2 functions to regulate actin polymerization dynamics, indicating a role in promoting motility or invasiveness of prostate cancer cells. These findings indicate a noncanonical mechanism by which EZH2 promotes cancer metastasis and invasiveness.

## 7. Perspective

EZH2 plays pivotal roles in pathogenesis, notably cancer. The mechanisms-of-actions regarding EZH2 are increasingly appreciated to be complex. Despite advances, it remains to be determined whether the so-far-reported nonhistone targets represent the scope of EZH2’s methylome, which merits further investigation such as unbiased proteomics-based surveys before and after cell treatment with the EZH2 catalytic inhibitor. Likewise, despite much progress in revealing noncanonical interactions between EZH2 and various TF/coactivators for gene activation, the molecular underpinning of such associations remains unclear in many cases. Both direct and indirect interactions likely exist, which requires further studies. In addition, it is noteworthy to mention that some of the above-covered findings might be complicated by both primary and secondary effects brought out by EZH2 manipulations, which are hard to tease apart in some cases, making difficult to ascribe the phenotypes to EZH2’s noncanonical role. Furthermore, it remains a puzzle why such a complicated functionality centering on EZH2 evolves, with both gene repression and activation effects clearly observed in same cells. Is it possible that it provides a mechanism for cells to sense assembly of PRC2, or that it might be a way to fine-tune PRC2-mediated repression? Clearly, cancer cells frequently gain genetic alterations, leading to increased EZH2 levels, and thus increasingly rely on EZH2’s noncanonical functions for malignant growth. A better understanding of the above issues regarding EZH2 and its context-dependent regulation in future shall help develop a more specific means for targeting cancer versus normal cells.

Proteolysis-targeting chimeras (PROTACs) emerge as an attractive therapeutic agent [86,87,88]. PROTAC represents a type of heterobifunctional small-molecule with one small-molecule binding to protein-of-interest (for example, EZH2) and the other moiety binding to cellular E3 ligase, which thereby leads target degradation via the proteasome. Two recent papers reported the EED-targeting PROTAC, which binds EED and recruits VHL for inducing degradation of PRC2 in cells [89,90]; an EZH2-selective degrader (not a PROTAC) employed a hydrophobic-tagging approach for depleting cellular EZH2, which shows early promise [29]. However, further studies are warranted to evaluate effects of these agents on EZH2’s noncanonical functions in cancer cells. As EZH2’s noncanonical actions often operate in a PRC2(EED)-independent manner, the disclosed EED PROTAC is unlikely to fully fill in the current gaps. We favor a view that development of the next-generation EZH2 inhibitors that can target both its enzymatic and non-enzymatic functions shall provide a promising and more effective strategy of therapeutics. We remain optimistic of future development along these lines.

## Figures and Tables

**Table 1:** Summary of nonhistone substrates of EZH2, methylation site, and related biological function.

	Substrate	Site	Function
Nuclear substrate	JAIRD2	K116-me3, K116-me2	JARID2-K116me3 is bound by EED, an event that induces allosteric activation of PRC2 in ESCs [35]
	EZH2	K510me, K514me, K515me	Automethylation of EZH2 activates PRC2, potentiating its histone methyltransferase activity [36,37]
	EolA	K754-me	EolA-K754 methylation by PRC2:EZH2 negatively impacts on activity of the Elongin complex, leading to downregulation of target gene expression in ESCs [38]
	STAT3	K180-me3	Promotes activation of STAT3 in glioblastoma stem-like cells [39]
		K49-me2	Promotes activation of STAT3, in response to IL-6 in colon cancer cells [40]
	PCNA	K110-me3	Stabilizes the PCNA trimer and stabilizes its association with DNA polymerase δ [41]
	AR	Not defined	Enhances AR activity at the AR/EZH2 co-targeted genes in advanced prostate cancer cells [42]
	GATA4	K299-me	Reduces GATA4’s transcriptional activation potency by preventing its interaction with P300 [43]
	PLZF	K430-me	Promotes PLZF ubiquitination and degradation in natural killer T cells [44]
	RORα	K38-me	Promotes RORα ubiquitination and degradation in cancer [45]
Cytosolic substrate	Talin	K2454-me3	Disrupts Talin binding to F-actin in neutrophil and dendritic cell [46]

**Table 2:** Summary of EZH2′s noncanonical partners in cancer.

	Cofactor	Function
Acting as coactivator	AR	EZH2 enhances AR signaling at their co-targeted genes in advanced prostate cancer [42]
	ER	Together with ERα and β-catenin, EZH2 coactivates genes coregulated by estrogen and WNT signaling in ER-positive luminal-like breast cancer cells [32]
	β-catenin	EZH2 coactivates target genes of Wnt/β-catenin signaling in mammary epithelial cells [69], ER-positive breast cancer [32] and colon cancer [71]
	NF-κB (RelA and RelB)	EZH2 promotes NF-κB signaling in the ER-negative breast cancer cells [70]
	PAF and β-Catenin	EZH2, PAF, and β-Catenin coactivates Wnt target genes in colon cancer cells [71]
	TRIM28 and subunit of the SWI/SNF complex	EZH2 co-activates a set of stemness related transcripts to promote mammosphere formation by TNBC cells [76]
	FoxM1	EZH2 co-activates genes related to tumor cell invasion, as part of HIF1a-mediated response to hypoxia [72]
Acting as corepressor	N-myc/C-myc	EZH2:PRC2 involved in Myc target gene silencing [63,64]
	ER	EZH2 co-represses the NF-κB target genes in ER-positive breast cancer cells [70]
